# Surveillance of abdominal aortic aneurysm using accelerated 3D non-contrast black-blood cardiovascular magnetic resonance with compressed sensing (CS-DANTE-SPACE)

**DOI:** 10.1186/s12968-019-0571-2

**Published:** 2019-10-28

**Authors:** Chengcheng Zhu, Lizhen Cao, Zhaoying Wen, Sinyeob Ahn, Esther Raithel, Christoph Forman, Michael Hope, David Saloner

**Affiliations:** 1 Department of Radiology and Biomedical Imaging, UCSF, 4150 Clement Street, San Francisco, CA 94121 USA; 2 Department of Radiology, Xuanwu Hospital, Beijing, China; 3 Department of Radiology, Beijing Anzhen Hospital, Capital Medical University, Beijing Institute of Heart, Lung and Blood Vessel Disease, Beijing, 100029 China; 4 Siemens Healthcare, San Francisco, CA USA; 5 Siemens Healthcare GmbH, Erlangen, Germany

**Keywords:** Abdominal aortic aneurysm, black blood CMR, Compressed sensing, Patient surveillance

## Abstract

**Background:**

3D non-contrast high-resolution black-blood cardiovascular magnetic resonance (CMR) (DANTE-SPACE) has been used for surveillance of abdominal aortic aneurysm (AAA) and validated against computed tomography (CT) angiography. However, it requires a long scan time of more than 7 min. We sought to develop an accelerated sequence applying compressed sensing (CS-DANTE-SPACE) and validate it in AAA patients undergoing surveillance.

**Methods:**

Thirty-eight AAA patients (all males, 73 ± 6 years) under clinical surveillance were recruited for this study. All patients were scanned with DANTE-SPACE (scan time 7:10 min) and CS-DANTE-SPACE (scan time 4:12 min, a reduction of 41.4%). Nine 9 patients were scanned more than 2 times. In total, 50 pairs of images were available for comparison. Two radiologists independently evaluated the image quality on a 1–4 scale, and measured the maximal diameter of AAA, the intra-luminal thrombus (ILT) and lumen area, ILT-to-muscle signal intensity ratio, and the ILT-to-lumen contrast ratio. The sharpness of the aneurysm inner/outer boundaries was quantified.

**Results:**

CS-DANTE-SPACE achieved comparable image quality compared with DANTE-SPACE (3.15 ± 0.67 vs. 3.03 ± 0.64, *p* = 0.06). There was excellent agreement between results from the two sequences for diameter/area and ILT ratio measurements (ICCs> 0.85), and for quantifying growth rate (3.3 ± 3.1 vs. 3.3 ± 3.4 mm/year, ICC = 0.95.) CS-DANTE-SPACE showed a higher ILT-to-lumen contrast ratio (*p* = 0.01) and higher sharpness than DANTE-SPACE (*p* = 0.002). Both sequences had excellent inter-reader reproducibility for quantitative measurements (ICC > 0.88).

**Conclusion:**

CS-DANTE-SPACE can reduce scan time while maintaining image quality for AAA imaging. It is a promising tool for the surveillance of patients with AAA disease in the clinical setting.

**Electronic supplementary material:**

The online version of this article (10.1186/s12968-019-0571-2) contains supplementary material, which is available to authorized users.

## Background

Abdominal aortic aneurysm (AAA) is a common condition and is associated with high mortality [1]. Currently, clinical management of AAA is based on the maximal diameter, and AAAs with either a diameter > 5.5 cm or rapid growth (> 1 cm/year) are candidates for intervention [2]. The majority of patients with smaller AAAs (< 5.5 cm) are followed with surveillance imaging. Ultrasound (US) is most commonly used for AAA surveillance; however, it is limited by significant inter-operator variability [3]. Computed tomography (CT) angiography (CTA) and non-contrast CT have excellent image quality, but require ionizing radiation and iodinated contrast, which is undesirable for repeated imaging.

Recently, a 3D non-contrast black-blood cardiovascular magnetic resonance (CMR) technique (DANTE-SPACE [4]) was developed for AAA imaging that provides high resolution and AAA diameter measurement that is comparable to the gold-standard CTA [5]. However, this technique requires a scan time of longer than 7 min. Scans of this length are poorly tolerated by patients and are prone to patient motion.

Compressed sensing (CS) has been used to accelerate image acquisition using a nonlinear iterative reconstruction of sparsely undersampled *k*-space data [6, 7]. CS has also been successfully applied to black-blood imaging of carotid plaque [8–10] and intracranial artery disease [11] and showed comparable image quality to fully sampled methods with significantly reduced scan times. However, previous studies were limited to volunteers or only included a small number of patients. The feasibility of CS use in the clinical setting, specifically, for aortic aneurysm wall imaging, has not been investigated. This study aims to evaluate whether an accelerated DANTE-SPACE technique using compressed sensing (CS-DANTE-SPACE) can be used for surveillance of patients with AAA disease with the benefit of scan time reduction.

## Methods

### Sequence design and optimization

A T_1_-weighted SPACE prototype sequence was used in this study. The flip angle train design and DANTE blood suppression parameters were adopted from a previous study [4].

The undersampling mask that was used has a Poisson-disc variable-density pattern with elliptical k-space coverage. The center of k-space is fully sampled to permit generation of a coil sensitivity map [12]. The acceleration was achieved by compressed sensing alone, without combination with generalized autocalibrating partially parallel acquisition (GRAPPA) and partial Fourier.

In the inline CS reconstruction used in this work as well as in [12], the following cost function was minimized:

$$ \hat{x}=\arg \underset{x}{\min}\frac{1}{2}\sum \limits_{n=1}^N{\left\Vert {y}_n-{F}_u\left({CSM}_n\odot x\right)\ \right\Vert}_2^2+\lambda {\left\Vert Wx\right\Vert}_1 $$

with $\hat{x}$ being the image to be reconstructed. The first term ensures data fidelity, *N* is the number of receive channels, *y*_*n*_ is the *n*-th raw data channel, *F*_*u*_ the undersampled Fourier transform, and *CSM*_*n*_ the respective coil sensitivity map. The second term promotes sparsity of the solution in the wavelet domain. It is therefore a reconstruction that combines CS and parallel imaging. *λ* is a regularization parameter that steers the tradeoff between smoothness and the reduction of noise and aliasing artifacts.

An acceleration factor of 5 (acquisition of 20% of k-space) was applied, based on the experience of CS-SPACE for intracranial vessel wall imaging [11]. The reconstruction parameters (regularization parameters and number of iterations) were optimized in 5 patients using the following steps. 1) While keeping the number of iterations at 40, the regularization parameters of 0.0005, 0.001, 0.002 and 0.004 were evaluated. 2) Once the optimized regularization parameter was determined, iterations of 10, 20 and 40 were evaluated. Two radiologists qualitatively rated images for each parameter setting and chose the best protocol.

### Study population and CMR acquisition

From October 2016 to April 2018, 38 patients (all males, 73 ± 6 years) with AAA disease (> 3 cm in diameter as identified on either ultrasound or CT) were recruited for high-resolution CMR. Patients with AAAs smaller than 5.5 cm were routinely monitored by repeated CMR every 6–12 months. Patient demographic data is shown in Table 1. Patient studies were conducted following human subject approval of the IRB of the University of California San Francisco. All subjects gave informed written consent for study participation.

**Table 1:** Patient demographics

	All (*n* = 38)
Age, mean ± SD	73 ± 6
Male, n, %	38 (100)
Height (cm)	176 ± 6
Weight (Kg)	82.9 ± 18.8
Body Mass Index	27.0 ± 6.1
Hypertension ^a^, n, %	28 (73.6)
DM ^b^, n, %	13 (34.2)
Smoking, n, %	22 (57.9)
CAD ^c^, n, %	15 (39.5)

a: Hypertension is defined as resting blood pressure > 140/90 mmHg;

b: Diabetes Mellitus;

c: Coronary Artery Disease

All CMR examinations were performed on a 3 T whole-body CMR systems (MAGNETOM Skyra, Siemens Healthineers, Erlangen, Germany) using an 18-channel body coil. For each scan, both DANTE-SPACE and CS-DANTE-SPACE were acquired in the same subject. DANTE-SPACE was acquired in the coronal plane during free breathing, covering the abdominal aorta from the renal arteries to the aortic bifurcation. Acquisition parameters were as follows: TR/TE = 800/20 ms; 32 × 32 cm^2^ field of view (FOV); 52 coronal slices with 1.3 mm slice thickness; GRAPPA 2 in phase-encoding direction and 6/8 partial Fourier in slice-encoding direction and with elliptical k-space scanning (total acceleration of 2.9), echo train length 60; 1.3 mm isotropic resolution, 3.4 averages, scan time 7:10 min. DANTE parameters for blood suppression were similar to those in a previous study [4]. CS-DANTE-SPACE had similar acquisition parameters to those for DANTE-SPACE apart from the use of a CS k-space under-sampling pattern with an acceleration factor of 5. Scan time was reduced to 4:10 min (a 41.4% reduction).

### Image analysis

Images were anonymized with a number code and were randomly sorted to avoid bias. Both reviewers were blinded to the imaging sequences and patient clinical information.

Two radiologists independently assigned qualitative scores to the image quality by reviewing the image quality using multi-planar reconstructions (MPR) in Horos software (ver 2.3.0):

- Score 1: Lumen and aneurysm wall were not seen clearly; non-diagnostic image quality.
- Score 2: Lumen and aneurysm wall were seen over most of the cross-section; diagnostic image quality.
- Score 3: Lumen and aneurysm wall were seen clearly with only mild blurring/artifacts; good image quality.
- Score 4: Lumen and aneurysm wall confidently delineated with sharp boundaries; excellent image quality.

Patients with poor image quality (score 1) were excluded from the analysis.

The two radiologists also independently measured the maximal diameter of the AAAs from MPR images [5]. Average growth rate (mm/year) of AAA was calculated as (AAA diameter at the latest time point – AAA diameter at baseline) / follow-up duration (years).

At the maximal diameter location of AAA, the intra-luminal thrombus (ILT) and lumen areas and signal intensities were also recorded. The adjacent psoas muscle signal intensity was recorded and the ILT-to-muscle signal intensity ratio was calculated. The ILT-to-lumen contrast ratio was also calculated. ILT was designated as bright if ILT-to-muscle signal ratio was larger than 1.2 as reported in previous studies [13, 14]. Four types of ILT were identified as previously described: Type 1 dominantly bright; Type 2 mixture of bright and iso-intense ILT; Type 3 all iso-intense ILT; Type 4 no ILT. Two reviewers blinded to the sequences used independently identified the ILT types on both CS-DANTE-SPACE and DANTE-SPACE images.

The sharpness of the aneurysm inner/outer boundaries was quantified based on the previously defined method [15]. Sharpness was quantified in two orthogonal directions across the aneurysm (left to right and anterior to posterior), and the average values were used.

In patients who had CTA within 3 months of CMR exam, the maximal diameter and ILT/wall area were measured by a reviewer on CTA using MPR. The diameter and area measurements made on CMR were compared to CTA as a reference standard.

For CMR scans, the impact of patient body-mass-index (BMI) and atrial fibrillation on image quality was evaluated. For the scans with sub-optimal image quality (score of 2), the major reasons were recorded (flow artifacts, motion artifacts, low signal to noise ratio etc).

### Statistical analysis

Normality assumptions were formally assessed using the Shapiro-Wilk test. Distributions were summarized using the median [inter-quartile range (IQR)] or the mean ± standard deviation (SD). Differences between the quantitative measurements using the two sequences and between the two reviewers were assessed using the Bland–Altman analysis and intra-class correlation coefficient (ICC). The mean of the pair-wise differences was reported as bias and the 95% limits of agreement (LOA = bias ±1.96 × SD). Measurement error was quantified by coefficient of variance (CV; CV = SD between measurements / mean× 100%). Cohen’s kappa (κ) was used to determine the agreement of ILT type identification. A *p*-value of less than 0.05 was considered significant. All *p*-values were 2-sided. GraphPad Prism 7.0a (GraphPad Software Inc., La Jolla, California, USA) and R Statistics (version 3.3.3, www.r-project.org; R Foundation for Statistical Computing, Vienna, Austria) were used for data analysis.

## Results

Among 38 patients recruited, all patients had diagnostic image quality (score ≥ 2). Nine patients received multiple CMR scans (2–4 times), with an average follow-up duration of 237 ± 118 days. In total, 50 pairs of DANTE-SPACE and CS-DANTE-SPACE were available for comparison.

**Fig. 1:**
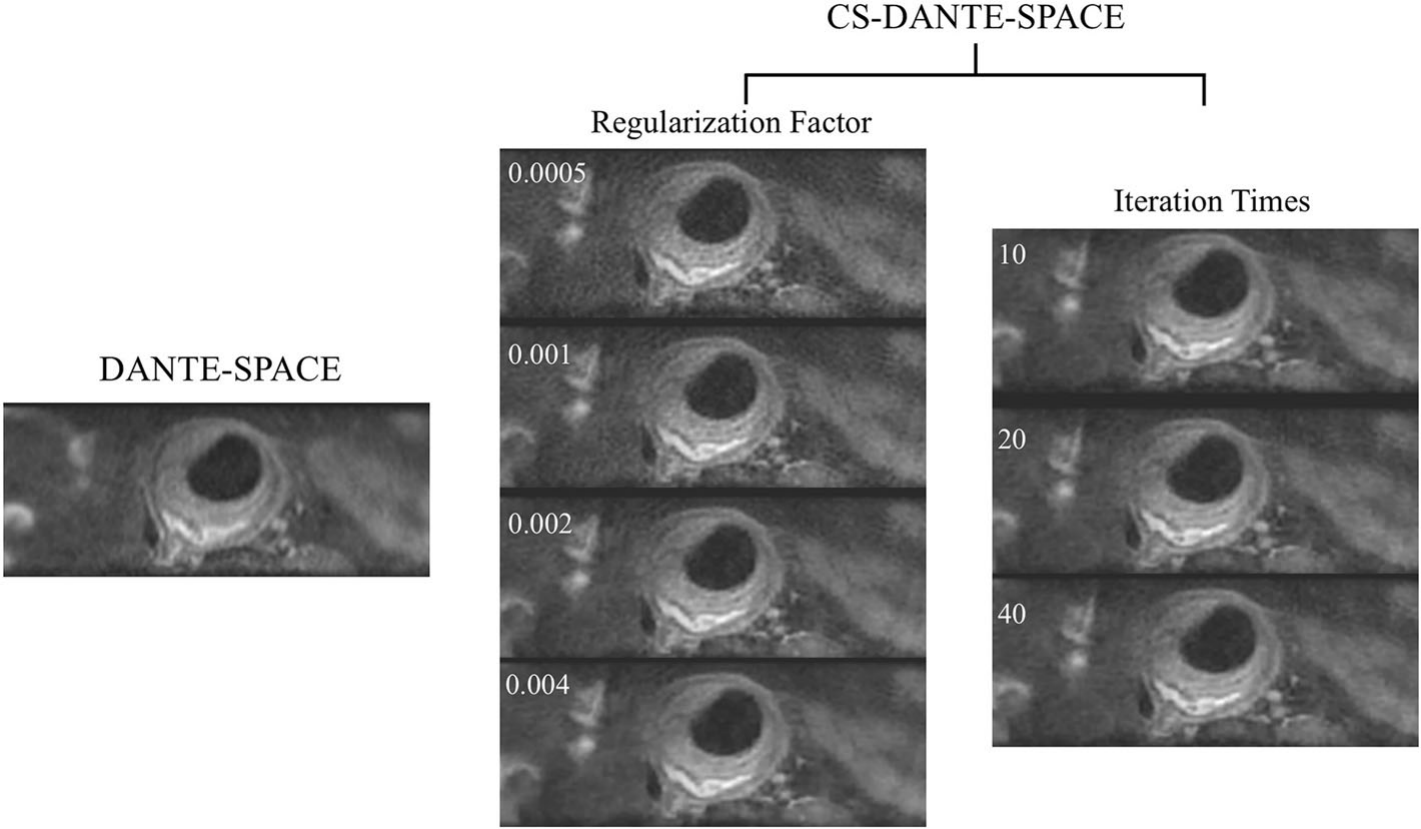
Optimization of reconstruction parameters for compressed sensing (CS) CS-DANTE-SPACE. A regularization factor of 0.002 had best image quality and 10 iteration times were sufficient

Reconstruction parameter optimization of CS-DANTE-SPACE was performed on images from 5 patients. Two readers reached consensus that a regularization factor of 0.002 had best image quality with a good balance between artifacts and signal to noise ratio (SNR), and found that there was no noticeable improvement in image quality for more than 10 iterations. Neither reader could detect differences among iterations of 10, 20, or 40. Sample images comparing different regularization factors and iteration times are shown in Fig. 1.

**Fig. 2:**
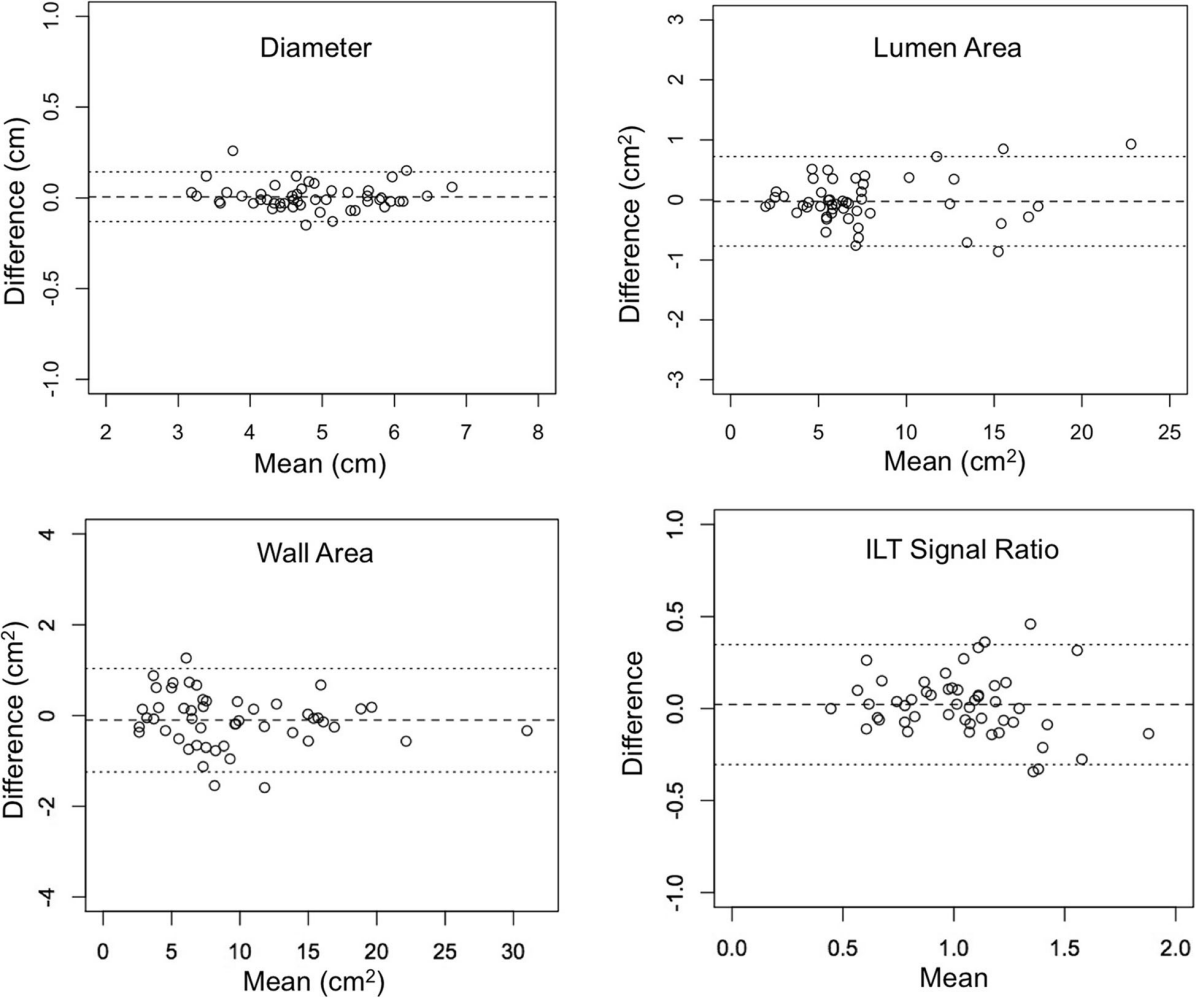
Bland-Altman plots for measurements from DANTE-SPACE and CS-DANTE-SPACE images (data from Reader 1 is shown). ILT = intraluminal thrombus

Qualitative and quantitative comparisons of the two sequences from reader 1 and reader 2 are shown in Table 2 and in the Additional file 2: Table S1. Corresponding Bland-Altman plots are shown in Fig. 2 and Additional file 1: Figure S1. CS-DANTE-SPACE has a comparable image quality score compared to DANTE-SPACE (3.22 ± 0.58 vs. 3.24 ± 0.62, *p* = 0.77). There was excellent agreement between data from the two sequences for diameter and area measurements (ICCs> 0.99) and good agreement for ILT ratio measurements (ICC = 0.779). There was no significant bias between results from the sequences for these measurements (*p* > 0.05). CS-DANTE-SPACE showed a higher ILT-to-lumen contrast ratio (*p* = 0.01) and higher lumen/wall boundary sharpness than DANTE-SPACE (*p* = 0.002).

**Table 2:** Qualitative and quantitative image quality assessment of DANTE-SPACE and CS-DANTE-SPACE. Results from reviewer 1 are shown. ILT = intraluminal thrombus

	DANTE- SPACE	CS-DANTE -SPACE	*P* value	ICC	Bias (LOA)	CV (100%)
Maximal Diameter (cm)	4.85 ± 0.87	4.84 ± 0.88	0.55	0.997	0.01(− 0.13, 0.14)	1.4
ILT/wall Area (cm^2^)	9.55 ± 5.72	9.62 ± 5.81	0.46	0.995	−0.07(−1.22, 1.10)	6.2
Lumen Area (cm^2^)	7.62 ± 4.46	7.64 ± 4.41	0.65	0.996	−0.02(− 0.77,0.72)	5.0
ILT signal ratio	1.05 ± 0.29	1.03 ± 0.32	0.35	0.851	0.02(−0.30,0.35)	16.0
Contrast Ratio	2.50 ± 0.91	2.95 ± 0.98	< 0.001	NA	NA	NA
Sharpness-inner (mm^−1^)	0.46 ± 0.53	0.52 ± 0.57	0.04	NA	NA	NA
Sharpness-outer (mm^−1^)	0.42 ± 0.83	0.47 ± 0.64	0.02	NA	NA	NA
Sharpness-average (mm^−1^)	0.44 ± 0.55	0.50 ± 0.48	0.002	NA	NA	NA
Image quality score	3.22 ± 0.58	3.24 ± 0.62	0.766	N	NA	NA

**Fig. 3:**
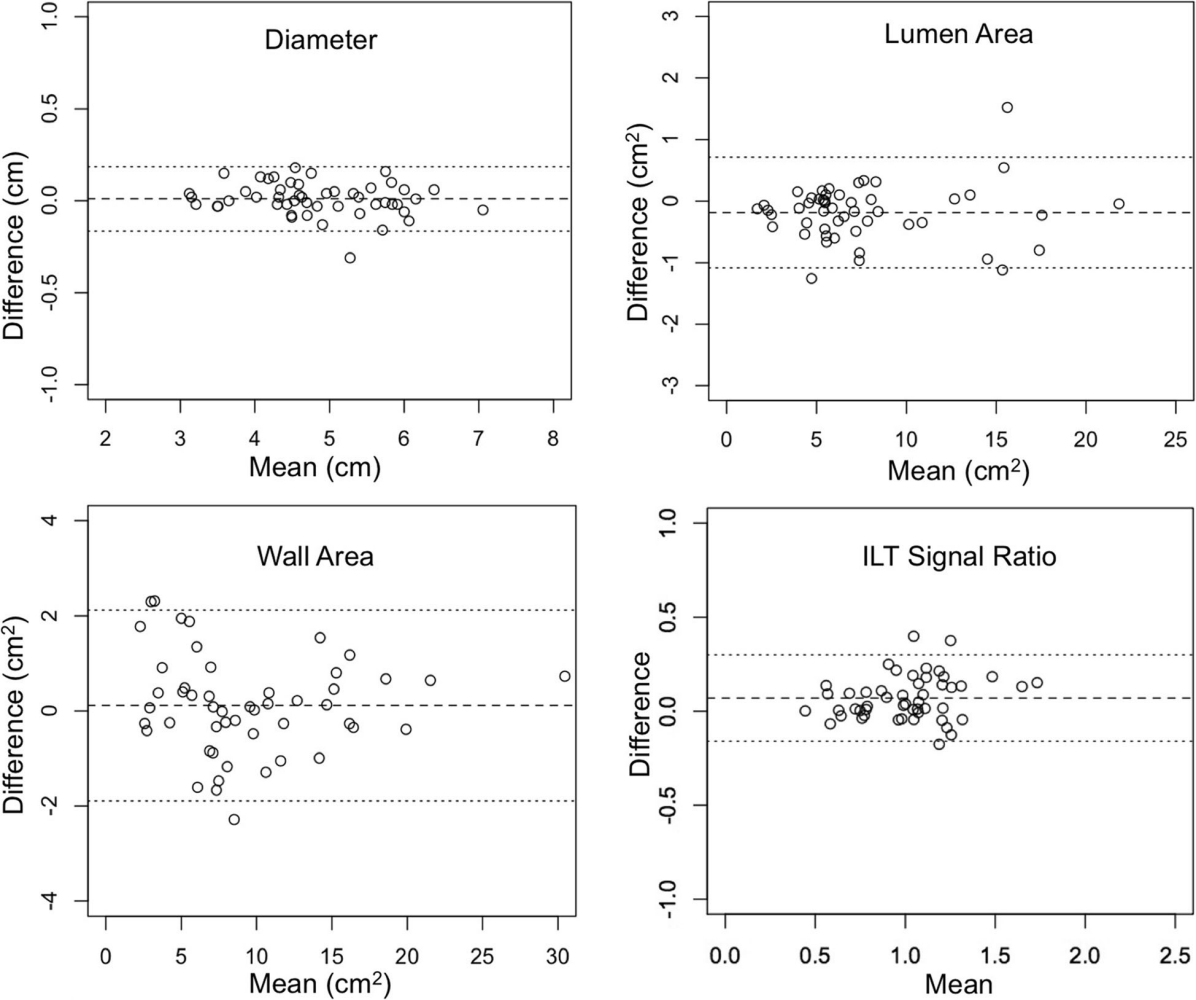
Bland-Altman plots showing inter-reader agreement of DANTE-SPACE measurements

**Fig. 4:**
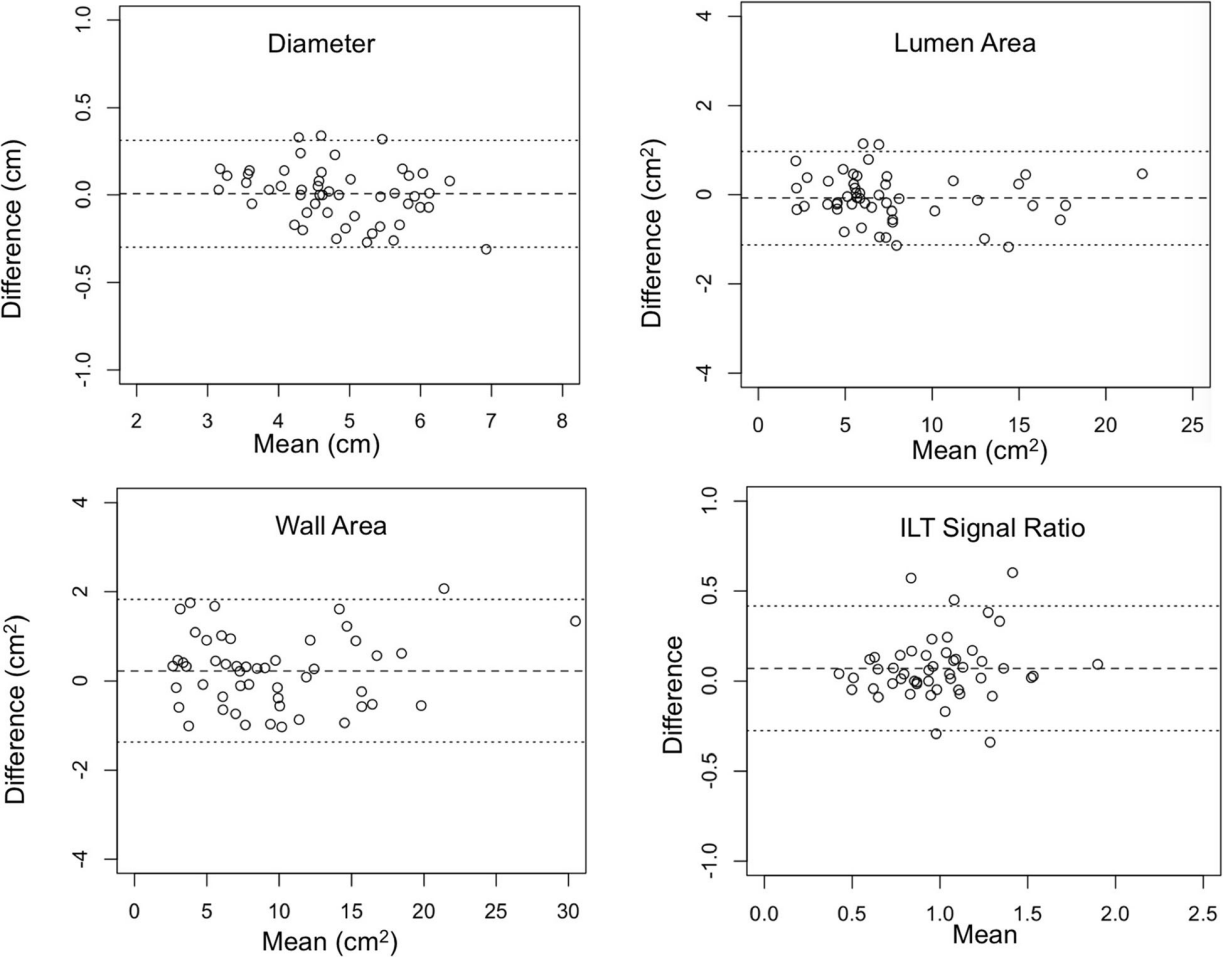
Bland-Altman plots showing inter-reader agreement of CS-DANTE-SPACE measurements

The inter-reader agreement for the measurements on DANTE-SPACE and CS-DANTE-SPACE is shown in Tables 3 and 4, and Bland-Altman plots are shown in Figs. 3 and 4. Results from the two radiologists’ reading were in excellent agreement for diameter/area measurements (ICC = 1.000 and 0.998) and ILT signal ratio measurements (ICC = 0.941). However, reader 2 tended to have slightly lower image quality scores than reader 1. While reader 1 didn’t observe a significant difference between image quality score between CS-DANTE-SPACE and DANTE-SPACE, reader 2 observed CS-DANTE-SPACE had better image quality than DANTE-SPACE (Additional file 2: Table S1). When pooled the image quality scores from the two readers, the image quality didn’t show a significant difference between CS-DANTE-SPACE and DANTE-SPACE (3.15 ± 0.67 vs. 3.03 ± 0.64, *p* = 0.06).

**Table 3:** Inter-reader agreement for quantitative measurements and qualitative image quality on DANTE-SPACE

	DANTE-SPACE Reader 1	DANTE-SPACE Reader 2	*P* value	ICC	Bias (LOA)	CV
Maximal Diameter (cm)	4.85 ± 0.87	4.84 ± 0.87	0.92	0.985	0.01(−0.30,0.30)	3.2
ILT/wall Area (cm^2^)	9.55 ± 5.72	9.44 ± 5.78	0.44	0.983	0.11(−1.90, 2.12)	10.8
Lumen Area (cm^2^)	7.62 ± 4.46	7.53 ± 4.52	0.31	0.990	0.09(−1.05, 1.22)	7.6
Contrast Ratio	2.50 ± 0.91	2.44 ± 0.94	0.18	0.944	0.06(−0.54,0.66)	12.4
ILT signal ratio	1.05 ± 0.29	0.98 ± 0.26	< 0.001	0.884	0.06(−0.16,0.30)	11.6
Image quality score	3.22 ± 0.58	2.84 ± 0.65	< 0.001	0.577	0.38(−0.73,1.49)	18.7

**Table 4:** Inter-reader agreement for quantitative measurements and qualitative image quality on CS-DANTE-SPACE

	CS-SPACE Reader 1	CS-SPACE Reader 2	*P* value	ICC	Bias (LOA)	CV
Maximal Diameter (cm)	4.84 ± 0.88	4.83 ± 0.92	0.76	0.985	0.01(−0.30,0.31)	3.2
ILT/wall Area (cm^2^)	9.62 ± 5.81	9.39 ± 5.72	0.06	0.989	0.23(−1.38,1.83)	8.6
Lumen Area (cm^2^)	7.64 ± 4.41	7.72 ± 4.49	0.31	0.993	−0.08(−1.13,0.97)	7.0
Contrast Ratio	2.95 ± 0.98	2.89 ± 0.93	0.50	0.944	0.06(−1.16,1.28)	21.4
ILT signal ratio	1.03 ± 0.32	0.95 ± 0.29	0.01	0.815	0.08(−0.28,0.42)	17.8
Image quality score	3.24 ± 0.62	3.06 ± 0.71	0.05	0.559	0.18(−1.05,1.41)	20.0

For the ILT type characterization, the two sequences had excellent agreement (kappa = 0.97 for reader 1 and 1.00 for reader 2) with good inter-reader reproducibility for both sequences (kappa = 0.74 and 0.71).

In 9 patients with longitudinal data, the average growth rate measured on DANTE-SPACE was (3.3 ± 3.1 mm/year), which was in good agreement with the growth rate measured on CS-DANTE-SPACE (3.3 ± 3.4 mm/year, ICC = 0.95).

Eight patients had close CMR and CTA (within 3 months) and one of them had CMR and CTA at two time points. A total of 9 paired CMR and CTA were compared. Both CS-DANTE-SPACE and DANTE-SPACE had good agreements for diameter and ILT/wall area measurements with CTA as a reference standard (diameter: ICC = 0.995 and 0.997; area: ICC = 0.931 and 0.940). For the one patient who had close CMR and CTA at two time points, the growth rate calculated by CMR and CTA were comparable (CS-DANTE-SPACE, 5.6 mm/year; DANTE-SPACE, 5.2 mm/year; CTA: 5.8 mm/year).

**Fig. 5:**
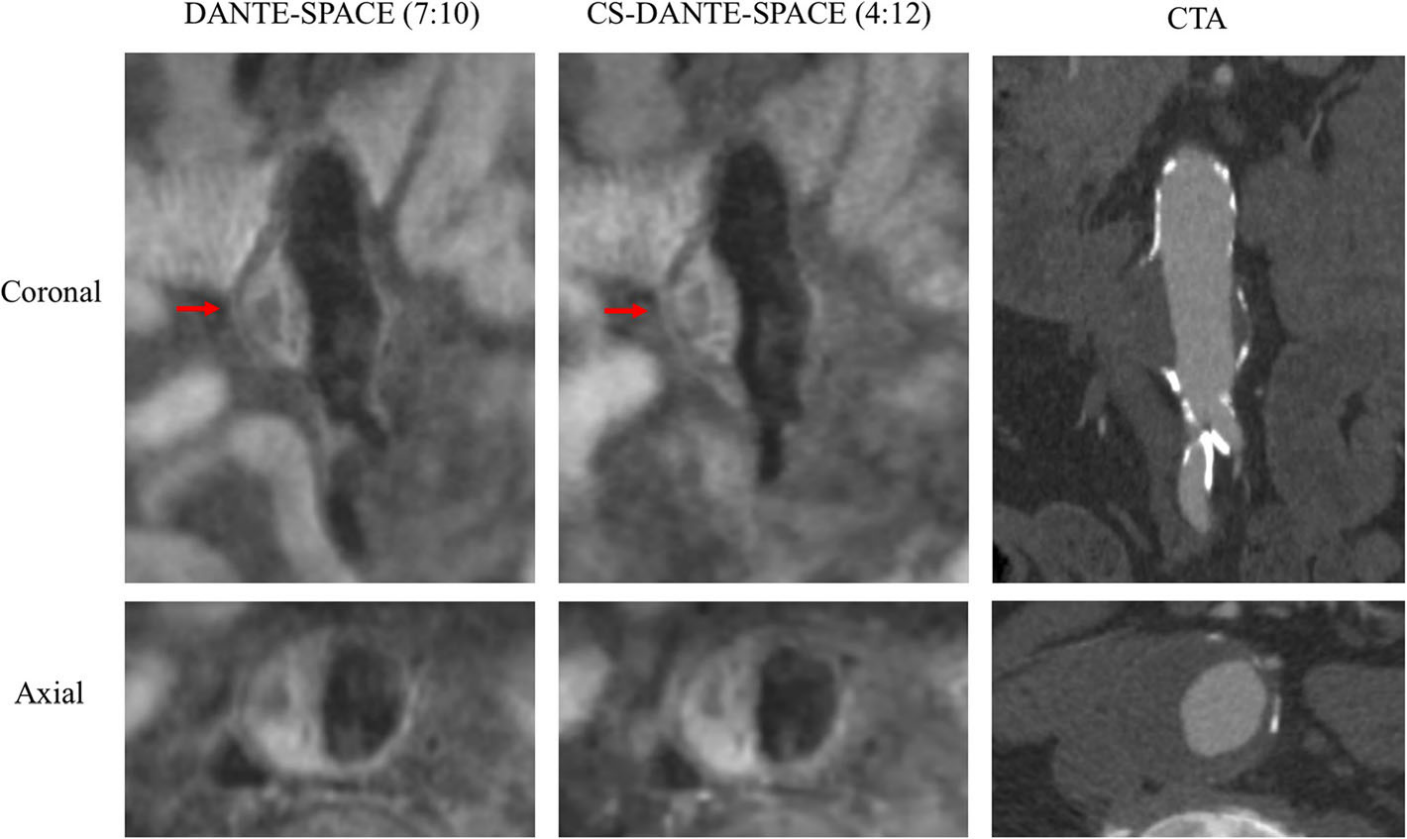
A patient with a 4.7 cm aneurysm. CS-DANTE-SPACE achieved comparable image quality with DANTE-SPACE, but with a 41% scan time reduction. Reference CTA 2 month before CMR is shown on the right. Red arrows show the level of maximal diameter. Heterogeneous intraluminal thrombus (ILT) was demonstrated in both sequences. A sharper image was noted for CS-DANTE-SPACE, possibly due to the reduced motion artifacts because of shorter scan time

**Fig. 6:**
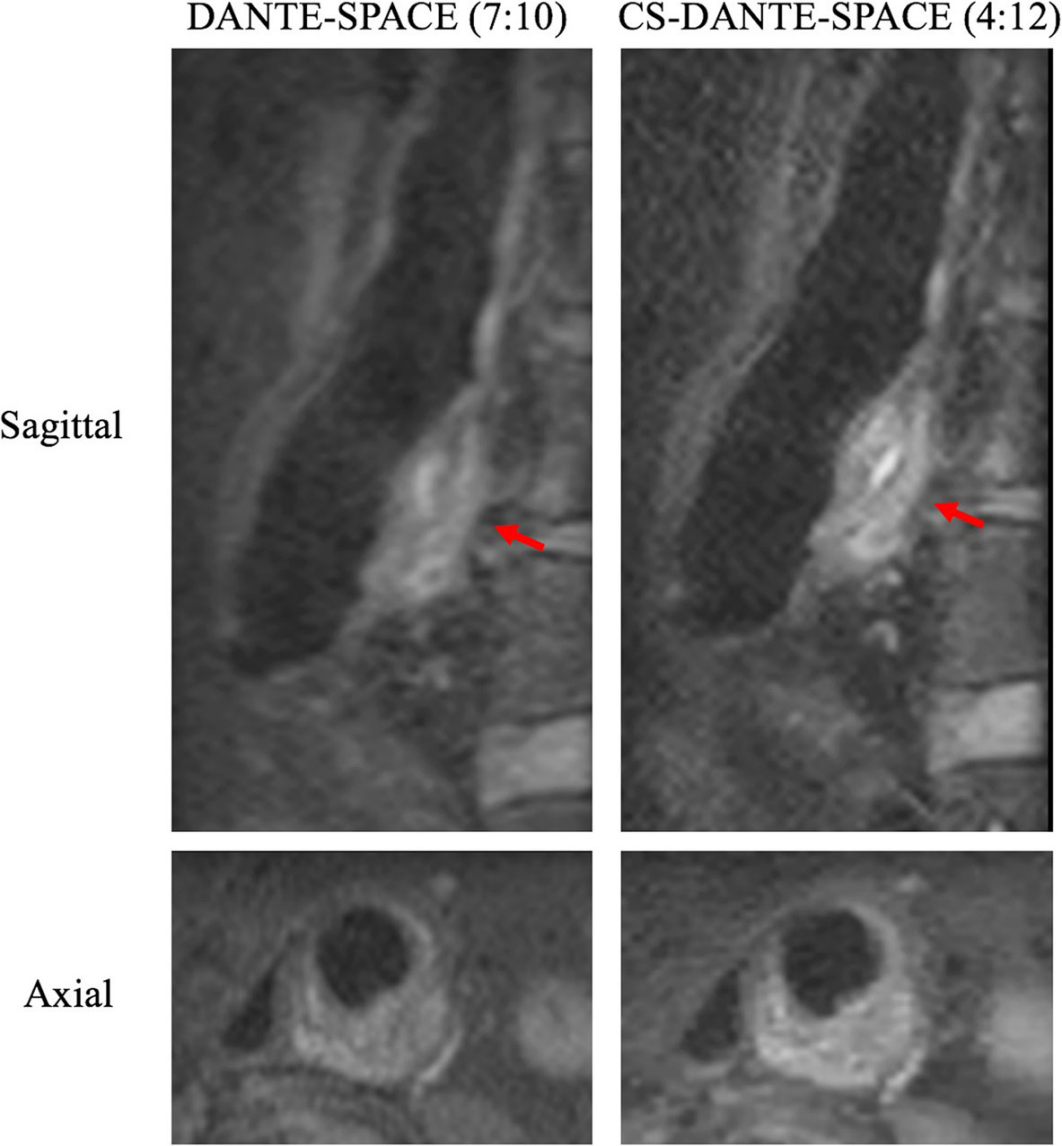
A patient with a 4.7 cm aneurysm. CS-DANTE-SPACE achieved comparable image quality with DANTE-SPACE, but with a 41% scan time reduction. CS-DANTE-SPACE has slightly higher sharpness and contrast ratio than DANTE-SPACE. Red arrows show the level of maximal diameter. Bright (fresh) intraluminal thrombus (ILT) was demonstrated in both sequences

Sample images of DANTE-SPACE and CS-DANTE-SPACE are shown in Figs. 5 and 6.

The image quality scores for obese patients (BMI > 30, *n* = 10) and non-obese patients (BMI < 30, *n* = 28) were compared and there was no significant difference for either CS-DANTE-SPACE (3.50 ± 0.52 vs. 3.23 ± 0.67, *p* = 0.21) or DANTE-SPACE sequences (3.42 ± 0.67 vs. 3.19 ± 0.60 *p* = 0.30). There were three patients with atrial fibrillation included in this study who received five CMR scans. The image quality of these five scans tended to be lower than the patients without atrial fibrillation CS-DANTE-SPACE (2.60 ± 0.55 vs. 3.31 ± 0.60, *p* = 0.01) and DANTE-SPACE (2.80 ± 0.84 vs. 3.27 ± 0.54, *p* = 0.09). There were 5 scans with suboptimal image quality (score of 2). Of the 5 scans, 2 had strong motion artifacts, 1 had both motion and flow artifacts, and 2 had low SNR in the middle of the abdomen.

## Discussion

To our knowledge, this is the first study that shows that CS black-blood vessel wall imaging can be used in a clinical surveillance program for AAA patients. We demonstrated that the CS-DANTE-SPACE can achieve accurate and reproducible measurement of AAA diameter/area, growth rates and ILT types compared with DANTE-SPACE as a reference, while reducing the scan time by more than 40%.

CS has been used to accelerate black-blood vessel wall imaging in previous studies [8–11]. Yuan et al. used a combination of parallel imaging and CS with 3D fast-spin-echo readout for carotid vessel wall black-blood T2 mapping and found good correlation with traditional 2D methods. However, they only evaluated their technique in six patients, and an acceleration factor of only three was achieved [10]. Makhijani et al. used a hidden Markov tree model-based compressed sensing method in a gradient echo black-blood sequence (MERGE) for carotid plaque imaging in a group of six patients [8]. They found good image quality and quantitative measurements with a 4.5-fold acceleration compared to non-accelerated scans. Zhu et al. evaluated CS black-blood SPACE sequence in 10 patients with intracranial vascular disease and achieved an acceleration factor of 5 without compromising image quality [11]. All these previous studies were conducted with limited sample size (number of patients < 10) and were not adequately evaluated in the clinical setting or for longitudinal studies. This study evaluated the utility of CS in patients under clinical surveillance program and provided clinically relevant results indicating that the CS method can be readily used in a clinical setting.

The use of non-contrast black-blood CMR for AAA surveillance has several advantages compared with other imaging modalities: 1) it has excellent accuracy and reproducibility which is comparable to gold standard CTA [5]; 2) it does not require ionizing radiation and/or iodinated contrast comparing to CTA or non-contrast CT; 3) it can characterize ILT composition which is a novel marker for AAA progression [13, 14] while ultrasound and CT cannot. However, it has disadvantages that include long scan time, higher cost, and limited availability. Our proposed method can significantly reduce the CMR scan time resulting in less motion sensitivity, improved patient comfort, and hence the likelihood of needing fewer repeated studies. It may therefore reduce associated costs and also facilitate the use of non-contrast CMR for AAA surveillance. Given that most AAAs are small, and there are many patients who have surveillance imaging, the impact of a reduction in scan time and cost is substantial.

CTA is the preferred method for AAA pre-surgical planning. As the proposed CS-DANTE-SPACE method shows clearly the geometry of AAA lumen and outer wall/ILT, it can also be potentially used for AAA intervention planning together with contrast enhanced or non-contrast enhanced CMR angiography [16, 17]. CMR angiography can be used to evaluate the femoral and iliac arteries’ diameters for endovascular treatment planning given the extended coverage. For the evaluation of vascular calcification, CT is still the gold standard, and novel CMR techniques including susceptibility weighted imaging [18] may also provide a good characterization of vessel wall calcification. The utility of these CMR based methods for AAA pre-surgical planning needs to be validated in future studies against gold standard CTA.

AAA disease is most prevalent in males with age > 65 years [1]. In the elderly population, a reduction in scan time can improve patient comfort and increase the success rate of quality data acquisition. Image quality can be potentially improved with reduced motion artifacts. In this study, we found the sharpness of CS-DANTE-SPACE was improved compared with SPACE, possibly due to the fact that the shorter scan time reduced the chance of motion contamination. Despite these improvement, there were still several patients with sub-optimal image quality (score of 2) with the presence of motion artifacts. No respiratory gating was used for black-blood CMR of AAA [4], due to: 1) the AAAs are largely immobilized by the spine with no significant motion, and 2) the use of respiratory gating will increase the scan time significantly by a factor of 2–3. However, the abdominal motion surrounding the AAA, especially in phase-encoding directions can still generate motion artifacts that propagate through the region of the AAA. One possible solution to further reduce motion artifact is to use radial k-space sampling (for example, stack of stars) together with gradient echo based sequences, which is inherently motion-robust and shows high promise in body imaging [19] and black-blood carotid vessel wall imaging [20]. The combination of compressed sensing and radial acquisition [19] can also be explored in future studies to reduce both the scan time and motion artifacts for AAA imaging. The performance of dedicated motion compensation techniques for abdominal imaging such as respiratory bellows, and navigators or self-gating [21, 22] should also be evaluated for black blood AAA imaging in future studies.

We also observed that CS-DANTE-SPACE had slightly better contrast to noise ratio (CNR) than DANTE-SPACE. While the echo train length and echo spacing were identical for the two sequences, the k-space under-sampling patterns were different (partial Fourier + GRAPPA vs. Poisson disk). Poisson disk has an uneven under-sampling pattern: higher density of points in the center than the edge in k-space [11], while GRAPPA has an even under-sampling of k-space except central reference lines. When a radial-like train path was used for T_1_-weighted SPACE [23], central part of k-space accounted for the majority of image contrast. Because the echo signal decreased with increased echo number in the train, the difference in k-space under sampling also induced a shift of the train towards the center, resulting in slight difference in image contrast, point spread function and blood suppression. These differences can be further studied in more details through theoretical simulations and phantom validations, which is, however, out of the scope of this study.

Electrocardiogram (ECG) triggering was not used in our study to simplify the patient set up. If the ECG triggering was used and the image acquisition was in the systolic phase (when flow was fast), the flow suppression could be further improved. In such situation, the variable R-R intervals within the patient and across different patients led to variable TR values (inconsistent T1 weighting), which was not an ideal condition for quantifying ILT signal intensity ratio, but it won’t affect maximal diameter quantification. By averaging, the motion and flow artifacts were suppressed, and also the SNR was improved, which was critical for such high isotropic resolution imaging.

There are several limitations of this study. First, it was a single-center study, and there were only a limited number of patients with longitudinal data. Future multi-center longitudinal studies with a larger patient cohort are needed to validate the feasibly of this technique. Second, only a small number of patients had CTA as a reference standard. However, the DANTE-SPACE sequence has previously been compared with CTA, showing excellent agreement [5]. Third, black blood CMR is not as easily visualized as bright blood techniques where maximal intensity projection (MIP) and volume rendering (VR) can be easily performed. MPR is normally used for black blood CMR. The use of an automatic segmentation method can help the 3D geometry reconstruction and visualization of AAA [24]. Fourth, no respiratory gating was used in this study and for the AAAs with tortuous geometry that were remote to the spine, strong motion artifacts could present. Fifth, we did not compare different CS undersampling patterns and reconstruction methods. Novel CS methods may further improve its utility for AAA surveillance and need to be evaluated in future studies.

## Conclusion

CS black-blood CMR (CS-DANTE-SPACE) can reduce scan time while maintaining image quality in AAA imaging. It is a promising tool for the surveillance of patients with AAA disease in the clinical setting.

## Additional files

Additional file 1: **Figure S1.** Bland-Altman plots for measurements from DANTE-SPACE and CSDANTE-SPACE images (data from Reader 2 is shown). (TIF 547 kb)

Additional file 2: **Table S1.** Qualitative and quantitative image quality assessment of DANTE-SPACE and CS-DANTE-SPACE. (Reader 2). (DOCX 15 kb)

## Data Availability

All data generated or analyzed during this study are included in this published article.

## Abbreviations

AAA: Abdominal aortic aneurysms
BMI: Body mass index
CMR: Cardiovascular magnetic resonance
CNR: Contrast-to-noise ratio
CS: Compressed sensing
CT: Computed tomography
CTA: Computed tomography angiography
ECG: Electrocardiogram
FOV: Field of view
ILT: Intraluminal thrombus
MIP: Maximum intensity projection
MPR: Multiplanar reconstruction
SNR: Signal-to-noise ratio
TE: Echo time
TR: Repetition time
US: Ultrasound
VR: Volume rendering

## References

[1] Kent KC, Zwolak RM, Egorova NN (2010). Analysis of risk factors for abdominal aortic aneurysm in a cohort of more than 3 million individuals. J Vasc Surg.

[2] LeFevre ML, Force USPST (2014). Screening for abdominal aortic aneurysm: U.S. preventive services task Force recommendation statement. Ann Intern Med.

[3] Beales L, Wolstenhulme S, Evans JA, West R, Scott DJ (2011). Reproducibility of ultrasound measurement of the abdominal aorta. Br J Surg.

[4] Zhu C, Haraldsson H, Faraji F (2016). Isotropic 3D black blood MRI of abdominal aortic aneurysm wall and intraluminal thrombus. Magn Reson Imaging.

[5] Zhu C, Tian B, Leach JR (2017). Non-contrast 3D black blood MRI for abdominal aortic aneurysm surveillance: comparison with CT angiography. Eur Radiol.

[6] Candes E, Romberg J, Tao T (2006). Robust uncertainty principles: exact signal reconstruction from highly incomplete frequency information. IEEE Trans Inform Theory.

[7] Lustig M, Donoho D, Pauly JM (2007). Sparse MRI: the application of compressed sensing for rapid MR imaging. Magn Reson Med.

[8] Makhijani MK, Balu N, Yamada K, Yuan C, Nayak KS (2012). Accelerated 3D MERGE carotid imaging using compressed sensing with a hidden Markov tree model. J Magn Reson Imaging.

[9] Li B, Dong L, Chen B (2013). Turbo fast three-dimensional carotid artery black-blood MRI by combining three-dimensional MERGE sequence with compressed sensing. Magn Reson Med.

[10] Yuan J, Usman A, Reid SA (2017). Three-dimensional black-blood T2 mapping with compressed sensing and data-driven parallel imaging in the carotid artery. Magn Reson Imaging.

[11] Zhu C, Tian B, Chen L (2018). Accelerated whole brain intracranial vessel wall imaging using black blood fast spin echo with compressed sensing (CS-SPACE). MAGMA.

[12] Fritz J, Raithel E, Thawait GK, Gilson W, Papp DF (2016). Six-fold acceleration of high-spatial resolution 3D SPACE MRI of the knee through incoherent k-space Undersampling and iterative reconstruction-first experience. Investig Radiol.

[13] Nguyen VL, Leiner T, Hellenthal FA (2014). Abdominal aortic aneurysms with high thrombus signal intensity on magnetic resonance imaging are associated with high growth rate. Eur J Vasc Endovasc Surg.

[14] Zhu C, Leach JR, Tian B (2019). Evaluation of the distribution and progression of intraluminal thrombus in abdominal aortic aneurysms using high-resolution MRI. J Magn Reson Imaging.

[15] Zhu C, Haraldsson H, Tian B (2016). High resolution imaging of the intracranial vessel wall at 3 and 7 T using 3D fast spin echo MRI. MAGMA.

[16] Francois CJ, Expert Panels on Vascular I, Interventional R (2018). ACR appropriateness criteria((R)) abdominal aortic aneurysm: interventional planning and follow-up. J Am Coll Radiol.

[17] Goshima S, Kanematsu M, Kondo H (2013). Preoperative planning for endovascular aortic repair of abdominal aortic aneurysms: feasibility of nonenhanced MR angiography versus contrast-enhanced CT angiography. Radiology.

[18] Yang Q, Liu J, Barnes SR (2009). Imaging the vessel wall in major peripheral arteries using susceptibility-weighted imaging. J Magn Reson Imaging.

[19] Feng L, Axel L, Chandarana H, Block KT, Sodickson DK, Otazo R (2016). XD-GRASP: Golden-angle radial MRI with reconstruction of extra motion-state dimensions using compressed sensing. Magn Reson Med.

[20] Kim SE, Roberts JA, Eisenmenger LB (2017). Motion-insensitive carotid intraplaque hemorrhage imaging using 3D inversion recovery preparation stack of stars (IR-prep SOS) technique. J Magn Reson Imaging.

[21] Vasanawala SS, Iwadate Y, Church DG, Herfkens RJ, Brau AC (2010). Navigated abdominal T1-W MRI permits free-breathing image acquisition with less motion artifact. Pediatr Radiol.

[22] Jin N, Lewandowski RJ, Omary RA, Larson AC (2009). Respiratory self-gating for free-breathing abdominal phase-contrast blood flow measurements. J Magn Reson Imaging.

[23] Mugler JP (2014). Optimized three-dimensional fast-spin-echo MRI. J Magn Reson Imaging.

[24] Wang Y, Seguro F, Kao E (2017). Segmentation of lumen and outer wall of abdominal aortic aneurysms from 3D black-blood MRI with a registration based geodesic active contour model. Med Image Anal.

