# Supplementary figures and images for: Surveillance of abdominal aortic aneurysm using accelerated 3D non-contrast black-blood cardiovascular magnetic resonance with compressed sensing (CS-DANTE-SPACE)
Source: J Cardiovasc Magn Reson. 2019 Oct 28;21:66. doi: 10.1186/s12968-019-0571-2 (PMC6816154; doi:10.1186/s12968-019-0571-2)

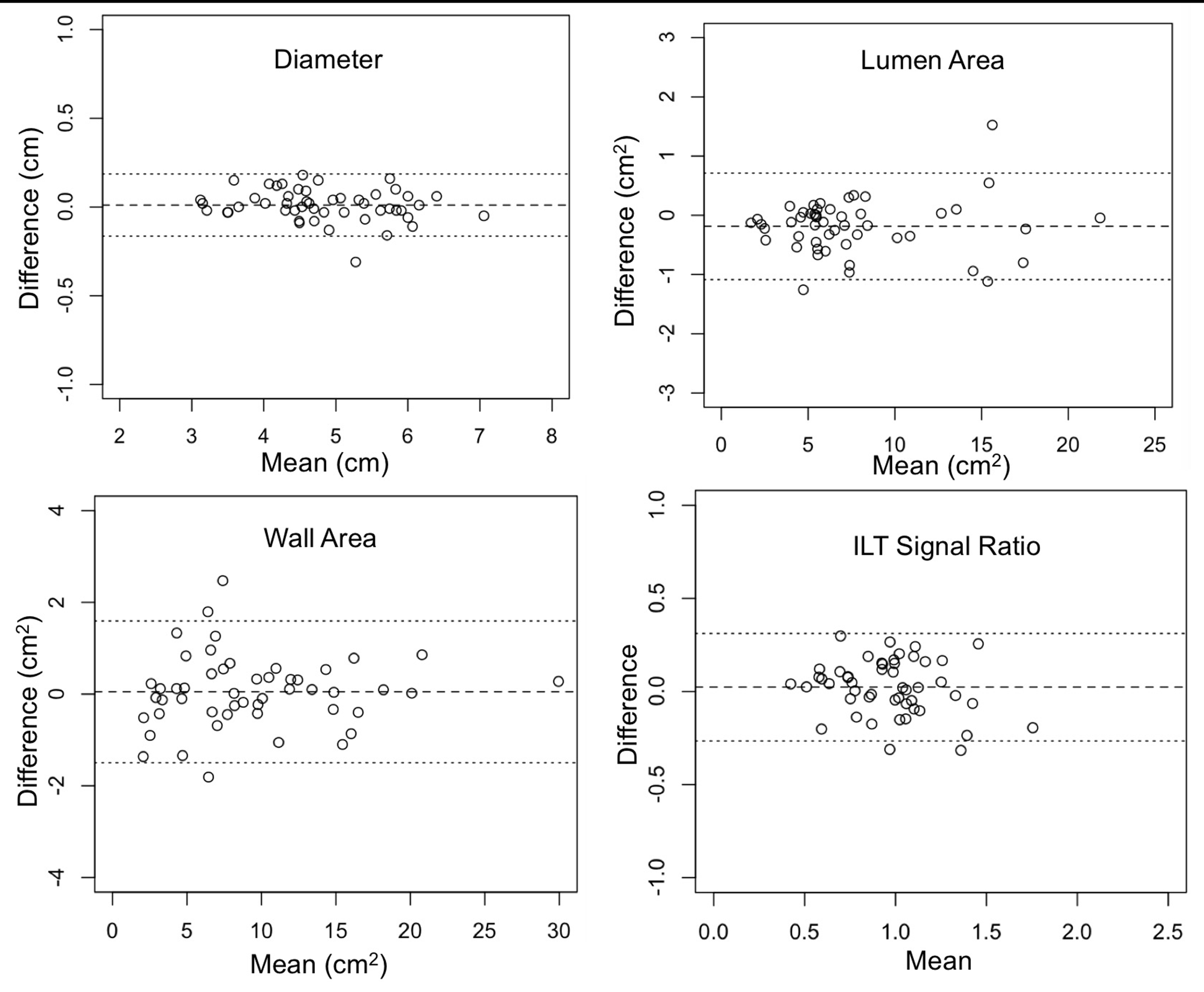

Supplement: Supplementary file 1 — Figure S1. Bland-Altman plots for measurements from DANTE-SPACE and CSDANTE-SPACE images (data from Reader 2 is shown). (TIF 547 kb) [file 12968_2019_571_MOESM1_ESM.tif]
