# Supplementary material for: Surveillance of abdominal aortic aneurysm using accelerated 3D non-contrast black-blood cardiovascular magnetic resonance with compressed sensing (CS-DANTE-SPACE)
Source: J Cardiovasc Magn Reson. 2019 Oct 28;21:66. doi: 10.1186/s12968-019-0571-2 (PMC6816154; doi:10.1186/s12968-019-0571-2)
Supplement: Supplementary file 2 — Table S1. Qualitative and quantitative image quality assessment of DANTE-SPACE and CS-DANTE-SPACE. (Reader 2). (DOCX 15 kb) [file 12968_2019_571_MOESM2_ESM.docx]

**Supplemental Table 1**. Qualitative and quantitative image quality assessment of DANTE-SPACE and CS-DANTE-SPACE. (Reader 2)

|  | DANTE-SPACE | CS-DANTE-SPACE | P value | ICC | Bias (LOA) | CV |
| --- | --- | --- | --- | --- | --- | --- |
| Maximal Diameter (cm) | 4.84±0.87 | 4.83±0.92 | 0.41 | 0.995 | 0.01(-0.16, 0.19) | 1.8 |
| ILT/wall Area (cm^2^) | 9.44±5.78 | 9.39±5.72 | 0.66 | 0.991 | 0.05(-1.50,1.60) | 8.4 |
| Lumen Area (cm^2^) | 7.53±4.52 | 7.72±4.49 | 0.006* | 0.994 | -0.19(-1.09,0.71) | 6.0 |
| ILT signal ratio | 0.98±0.26 | 0.95±0.29 | 0.27 | 0.860 | 0.03(-0.27,0.31) | 15.2 |
| Contrast Ratio | 2.44±0.94 | 2.89±0.93 | <0.001* | NA | NA | NA |
| Image quality score | 2.84±0.65 | 3.06±0.71 | 0.02* | NA | NA | NA |
